# Elevated level of Interleukin-35 in colorectal cancer induces conversion of T cells into iTr35 by activating STAT1/STAT3

**DOI:** 10.18632/oncotarget.12193

**Published:** 2016-09-22

**Authors:** Yanhui Ma, Lei Chen, Guohua Xie, Yunlan Zhou, Chaoyan Yue, Xiangliang Yuan, Yingxia Zheng, Weiwei Wang, Lin Deng, Lisong Shen

**Affiliations:** ^1^ Department of Laboratory Medicine, Xinhua Hospital, School of Medicine, Shanghai Jiao Tong University, Shanghai 200092, China; ^2^ Department of General Surgery, Xinhua Hospital, School of Medicine, Shanghai Jiao Tong University, Shanghai 200092, China

**Keywords:** interleukin-35, colorectal cancer, STAT1, STAT3

## Abstract

IL-35 is a novel heterodimeric and inhibitory cytokine, composed of interleukin-12 subunit alpha (P35) and Epstein-Barr virus -induced gene 3 (EBI3). IL-35 has been reported to be produced by a range of cell types, especially regulatory T cells, and to exert immunosuppressive effects via the STATx signaling pathway. In this study, we demonstrated that IL-35 expression was elevated in both serum and tumors in patients with colorectal cancer. IL-35 mainly expressed in CD4^+^ T cells in human colorectal cancer tumors and adjacent tissues. Increased IL-35 expression in tumor-adjacent tissues was significantly associated with tumor metastasis. IL-35 inhibited the proliferation of CD4^+^CD25^−^ T effector cells *in vitro* in a dose-dependent manner, and its suppression was partially reversed by applying IL-35-neutralizing antibodies. IL-35 treatment activated the phosphorylation of both STAT1 and STAT3 in human CD4^+^ T cells. Meanwhile, IL-35 induced a positive feedback loop to promote its own production. We observed that Tregs obtained from colorectal cancer patients were capable of inducing more IL-35 production. In addition, EBI3 promoter-driven luciferase activity was higher than that of the mock plasmid after IL-35stimulation. Thus, our study indicates that the high level of IL-35 in colorectal cancer promotes the production of IL-35 via STAT1 and STAT3, which suppresses T cell proliferation and may participate in tumor immunotolerance.

## INTRODUCTION

Colorectal cancer (CRC) is the third most common cancer in men and the second most common cancer in women worldwide [1–3]. The ability to evade immune destruction is one of the hallmarks of cancer [4], and regulatory T (Treg) cells are a critical sub-population of CD4^+^T cells for maintaining self-tolerance and preventing autoimmunity [5, 6]. Some studies have indicated that an increased number of regulatory T cells in the blood and tumors of colorectal cancer patients may result in an immune-compromised state against cancer. These studies suggest that strategies to overcome regulatory T cell activity may be beneficial in treatments for human colorectal cancer [7–9].

IL-35 as an inhibitory cytokine that was shown to be specifically produced by Treg cells in early studies, and is required for maximal suppressive activity in Tregs [10, 11]. But it has since been reported to be produced in B cells [12] and tumor cells (B lymphoma) [13] as well. IL-35 suppresses immune responses by expanding regulatory T cell development and suppressing Th17 cell development [10, 11]. Detectable levels of IL-35 have been reported in patients with chronic hepatitis [14], ulcerative colitis [10], and pancreatic ductal adenocarcinoma [15]. A mathematical model was used to show that IL-35 promoted tumorigenesis in colorectal cancer [16]. Because these results were based on a computer analysis, laboratory-based evidence for the role of IL-35 and/or iTr35 in cancer was needed to support their relationship and functions in humans.

In mice, IL-35 exerts its immunosuppressive role via EBI3 subunits, and by binding to its ligand, it downregulates the expression of IL-17, IL-22 and RORγt [17]. IL-35 also converts Foxp3^−^ CD4^+^ T cells into inducible Tregs (iTregs) and promotes the expression of IL-35 via a positive feedback loop to involve in immunoregulation [11]. *In vivo* results in mice confirmed that Tregs secreted IL-35 and induced conventional T cells (Tconv) to iTregs, called iTr35 cells, independent on IL-10 and TGF-β [11, 18]. However, the results supporting the association between human IL-35 and iTr35 cells have varied. One study showed that human Tregs inhibited the immune reaction mediated by IL-35 through inducing Tconv cells into iTr35 cells [11]. In addition, IL-35 suppressed the MAPK-AP-1 pathway in endothelial cell [19]. However, this process has not yet been further explored in human diseases.

The expression and regulation of IL-35 is regulated by its ligand and downstream signal pathway. Studies have reported that the IL-35 receptor (IL-35R) can be a heterodimer containing both IL-12Rβ2 and gpl30 (also IL-6 signal transducer, Il6st) and activate STAT 1/4, or can be homodimer containing either IL-12Rβ2 or gp130 and activate STAT1 or STAT4 separately [20]. Wang et al. [21] found that IL-35 in T cells was capable of activating STAT3 and STAT1/4, but the activation of STAT1 and STAT3 was mediated by a heterodimeric receptor composed of IL-12Rβ2 and IL-27Rα in B cells. These studies were performed in mice, however.

In this study, we found high levels of expression of IL-35 in serum and the tumor microenvironment in CRC patients that correlated with an increase in Tregs. IL-35 expression was assessed using immunofluorescence double staining and was found to be expressed mainly in CD4^+^ T cells in tumor and tumor adjacent tissues. CD25^−^CD4^+^ We found that IL-35 enhanced EBI3 mRNA expression and IL-35 protein levels in T cells and a decrease in Tconv proliferation, suggesting that they were induced to differentiate into iTr35 cells. Interestingly, our experiments in human CD4^+^ T cells, but not those performed in B cells, showed that rhIL-35 activated the phosphorylation of both STAT1 and STAT3. We speculate that IL-35R might be present in a diverse array of combinations, each of which might lead to different IL-35 activation signals in different species or cell types. Thus, our findings suggest that elevated IL-35 levels induced the generation of iTr35 cells, inhibited proliferation in T effector cells, and activated STAT1 and STAT3 heterodimers in human CD4^+^ T cells. These results may support the potential therapeutic role of IL-35 in colorectal cancer treatment.

## RESULTS

### An increase in Treg cells and IL-35 expression was detected in patients with colorectal cancer

**Figure 1:**
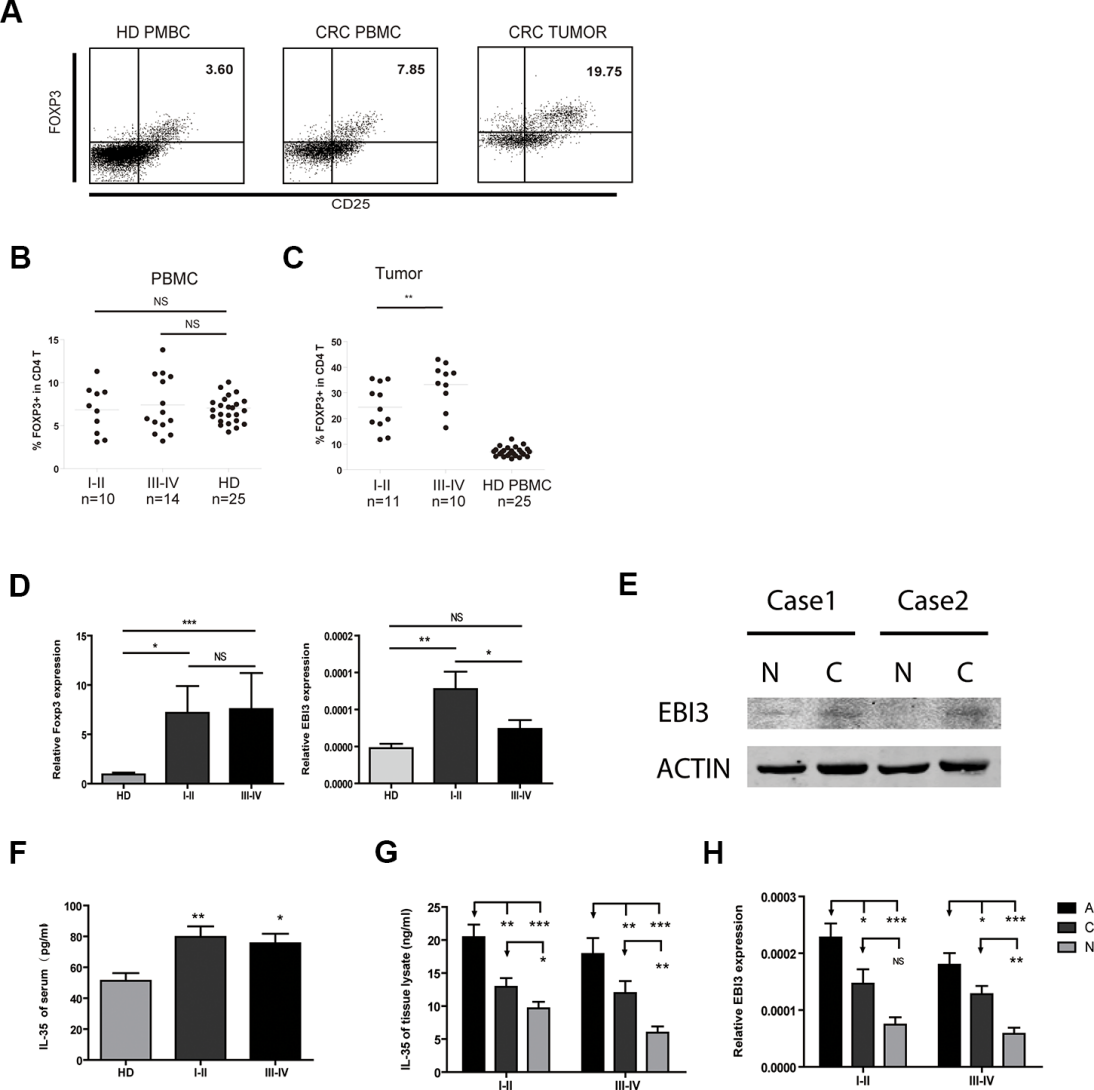
Increased Treg numbers and IL-35 levels in patients with CRC (**A**) Representative flow cytometric (FCM) dot plots showing Foxp3 and CD25 double-labeled CD4 T cells both in healthy donors (HD) and CRC patients. (**B** and **C**) Significantly increased proportions of Treg cells in CRC patients in early and advanced clinical stages compared to the proportions in healthy donors. (**D**) The mRNA expression levels of Treg-related transcription factors and the IL-35 subunit EBI3 in PBMCs. (**E**) Immunoblotting analyzed the EBI3 expression in normal (N) and cancer (C) tissue of patients with CRC. (**F**) Serum IL-35 concentrations of HD and CRC were detected by ELISA. (**G**) IL-35 concentrations in lysate supernatant of colorectal cancer (Dark Grey, C), adjacent (Black, A) and normal mucosal tissues (Light grey, N) were detected. (**H**) The mRNA expression levels of the IL-35 subunit EBI3 in PBMCs. Data represent the mean ± SEM. *p < 0.05, **p < 0.01, and ***p < 0.001 compared to the HD group.

To determine whether Treg cells and IL-35 are involved in the development of CRC, we first measured the number of circulating Treg cells in PBL using flow cytometry. We then defined the phenotype of the Treg cells as CD4^+^CD25^+^Foxp3^+^. The proportion of Tregs in the HDs ranged from 4.1% to 10.1%, while it ranged from 3.2% to 13.8% in the patients with CRC (Figure 1A and 1B). The frequency of Treg cells was higher in patients with CRC, but there was no significant difference between the groups. Treg cells were markedly increased in tumor tissues and were associated with clinical stage (I-II 24.4 ± 2.7% vs III-IV 33.3 ± 2.8%) (*P* < 0.05, Figure 1C). Interestingly, the proportion of Th17 cells in the total CD4^+^ T cells was higher in patients with CRC than in the HDs (Supplementary Figure S1A and S1B). Combined with the increased Foxp3 mRNA levels in CRC PBMC, EBI3 mRNA expression was upregulated in early stage of CRC (Figure 1D). To some extent, EBI3 expression might not be consistent with Foxp3 levels. Western Blot results showed that the protein expression of EBI3 was higher in cancer (C) than in normal tissues (N) (Figure 1E). High levels of IL-35 were expressed in the CRC serum as measured using ELISA (Figure 1F). IL-35 protein and mRNA expression levels were further investigated in lysate of cancer (C), cancer-adjacent (A) and normal (N) tissues both in early and advanced CRC patients. IL-35 protein was consistent with the increase in EBI3 mRNA expression in tissues (Figure 1G and 1H). Overall, the data show that Treg cells and IL-35 expression were increased in CRC patients.

### IL-35 was highly expressed in CD4^+^ T cells in tumor-adjacent tissues and was associated with tumor metastasis

**Figure 2:**
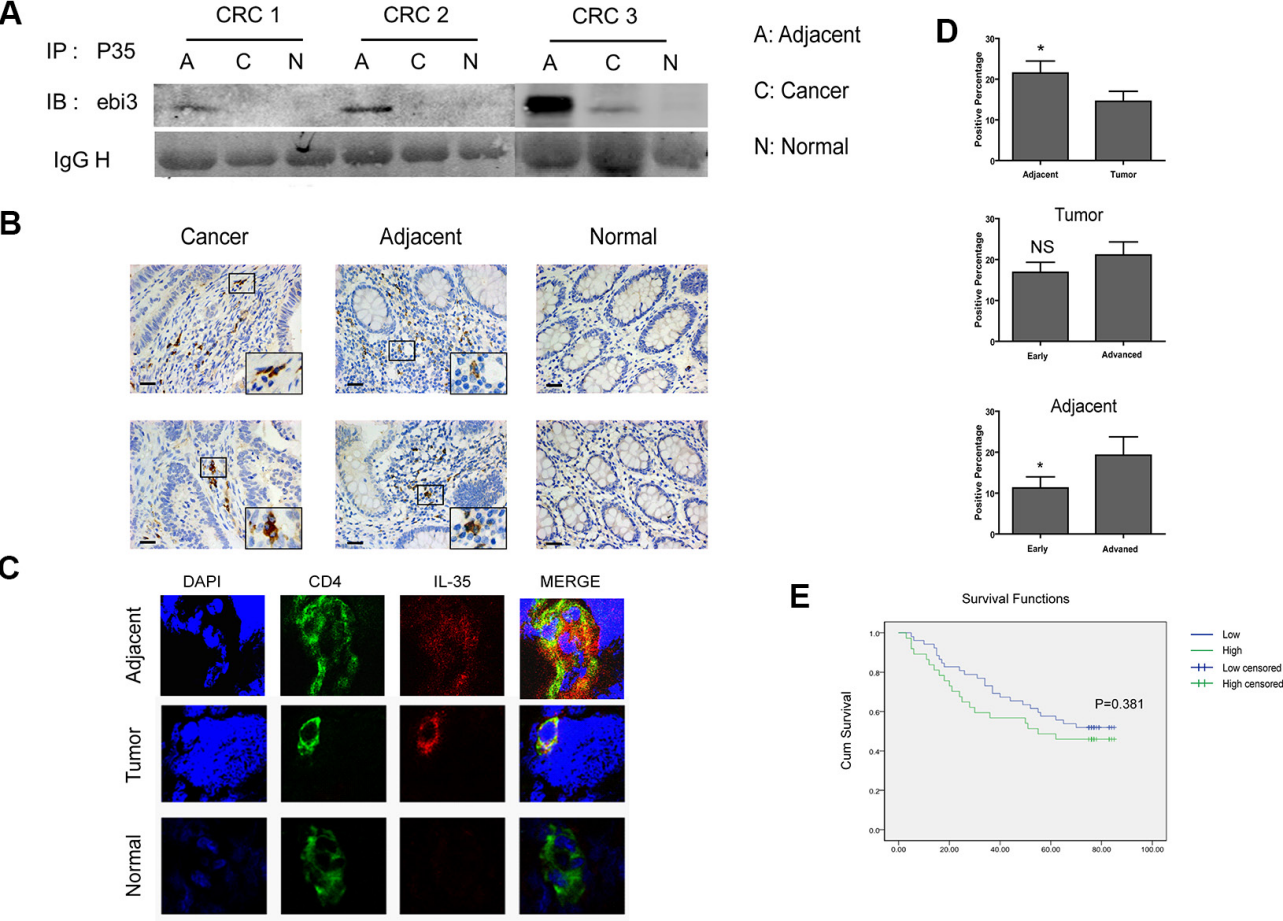
IL-35 is expressed in CD4^+^ T cells in tumor-adjacent tissues and is associated with tumor metastasis (**A**) Colorectal cancer (C), adjacent (A) and normal mucosal tissues (N) (200 mg) lysis supernatants were immunoprecipitated using anti-human IL-12A antibodies and then coupled to protein A/G-agarose beads. Proteins were resolved using SDS-PAGE, and the blots were then probed using monoclonal anti-human EBI3 antibodies. (**B**) Representative images of cells labeled for the cytoplasmic marker IL-35 using immunohistochemistry. IL-35 was detected using mouse anti-human IL-35 monoclonal antibodies that were diluted 1:300 as the primary antibody in tumor, tumor-adjacent and mucosal tissues from the same individual. Scale bars, 100 μm. (**C**) Representative images obtained using confocal microscopy showed CD4 (green) and IL-35 (red) expression in frozen tissue samples. Nuclei were stained with DAPI (blue). (**D**) The total scores were calculated as the intensity of staining for IL-35 plus the percentage of positive cells in tumor and tumor-adjacent tissues in patients with early and advanced stages. (**E**) A Kaplan-Meier curve was plotted and used to analyze overall survival in CRC patients based on the total scores (see above): low expression group (total score < 1) and high expression (total score ≥ 1) groups. Data represent the mean ± SEM. *p < 0.05.

We applied immunoprecipitation, immuno- histochemistry and immunofluorescence assays to further analyze the expression and localization of heterodimeric cytokine IL-35. Tissue lysis was immunoprecipitated with anti-human IL-12A and detected using anti-human EBI3 antibodies. We found high levels of IL-35 expression in tumors and tumor-adjacent tissues (Figure 2A and Supplementary Figure S1C). IL-35-positive cells were primarily lymphocytes in both tumors and tumor-adjacent tissues (Figure 2B), and IL-35 was especially highly expressed in CD4^+^ T cells (Figure 2C). The staining score for IL-35 was higher in adjacent tissues than in the tumors themselves and was associated advanced clinical stage tumors (Figure 2D, *P* < 0.05). However, higher IL-35 expression was not significantly associated with reduced overall survival in patients at follow-ups (Figure 2E, *P* > 0.05). While, its expression in adjacent tissues was correlated with distant metastasis (*P* = 0.007) (Table 2). As expected, IL-35 was expressed in CRC, and it was significantly associated with tumor metastasis.

**Table 1:** Correlations with IL-35 expression in tumor and tumor-adjacent tissues

		Tumor	Adjacent	Gender	Age	Pathological grading	Tumor size	T Stage	Lymphatic metastasis	Distant metastasis	TNM Stage
Tumor	Correlation Coefficient	1.000	.299**	−.021	−.209	.206	−.064	.048	.013	.028	.035
	Sig. (2-tailed)	/	.005	.845	.052	.053	.553	.660	.902	.794	.748
	N	89	85	89	87	89	87	87	89	88	87
Adjacent	Correlation Coefficient	.299**	1.000	.061	−.010	.062	−.139	−.076	.110	.291**	.109
	Sig. (2-tailed)	.005	/	.575	.929	.572	.207	.487	.311	.007	.322
	N	85	86	86	84	86	84	85	86	85	85

**Table 2:** Clinical characteristics of CRC patients

Characteristics	Colon Cancer *N* (%)			Rectal Cancer *N* (%)		
	STAGE		*P*	STAGE		*P*
	I-II	III-IV		I-II	III-IV	
N	38	39		22	26	
Age						
≤ 60	13 (34.21)	12 (30.77)	0.81	7 (31.82)	11 (42.31)	0.555
< 60	25 (65.79)	27 (69.23)		15 (68.18)	15 (57.69)	
Gender						
Male	13 (34.21)	20 (51.28)	0.169	13 (59.09)	14 (53.85)	0.776
Female	25 (65.79)	19 (48.72)		9 (40.91)	12 (46.15)	
Location						
Ascending colon	14 (36.84)	15 (38.46)	0.442	NA	NA	
Transverse colon	3 (7.89)	7 (17.95)		NA	NA	
Descending colon	9 (23.68)	4 (10.26)		NA	NA	
Ileocecal region	2 (5.26)	3 (7.70)		NA	NA	
Sigmoid colon	10 (26.32)	10 (25.64)		NA	NA	
Rectum	NA	NA		22 (100)	26 (100)	NA
Pathological type						
Tubular adenocarcinoma	31 (81.58)	28 (71.80)	0.728	16 (72.73)	21 (80.77)	0.544
Mucinous Adenocarcinoma	2 (5.26)	4 (10.26)		0(0)	0 (0)	
Papillary Adenocarcinoma	2 (5.26)	2 (5.13)		2 (9.09)	3 (11.54)	
Unknown	3 (7.89)	5 (12.82)		4 (18.18)	2 (7.69)	
Differentiation						
Poorly differentiated	2 (5.26)	6 (15.38)	0.345	2 (9.09)	2 (7.69)	0.281
Moderately Differentiated	34 (89.47)	31 (79.49)		18 (81.82)	24 (92.31)	
Well differentiated	2 (5.26)	2 (5.13)		2 (9.09)	0 (0)	

### Reconstituted human IL-35 inhibited CD4^+^ T cell proliferation

**Figure 3:**
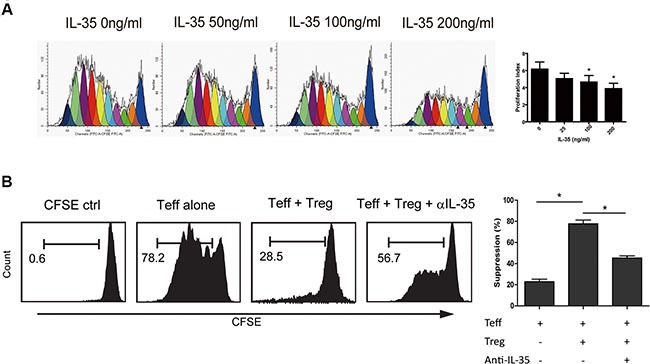
IL-35 suppressed CD4^+^ T cell proliferation (**A**) CFSE-labeled CD4^+^CD25^−^ T (1 × 10^5^ per well) cells obtained from HDs were incubated in a series of concentrations of rhIL-35 for 5 days and analyzed to determine the cell proliferation index using FACS and the appropriate software (ModFit LT; BD). (**B**) Using a transwell culture system (0.4 μm), as shown, CFSE-labeled CD4^+^CD25^−^ T cells (1 × 10^5^ per well) rom HDs were co-cultured with autologous CD4^+^CD25^+^ T cells (5 × 10^4^ per well) in the presence of IL-35-neutralizing antibodies. Cell proliferation was analyzed using FACS. The data shown are representative of three independent experiments. The results are shown as the mean ± SEM. *p < 0.05.

In previous *in vitro* studies, the suppression of IL-35 on T cell proliferation was reported to be dependent on IL-35 itself but not on IL-10 or TGF-β in mice. To determine the role of IL-35 in immunosuppression, we treated CFSE-labeled CD4^+^CD25^−^ T cells obtained from HD with a series of concentrations of rhIL-35 ranging from 0 ng/ml to 200 ng/ml for 5 days and then detected cell proliferation using FACS. IL-35 markedly inhibited T cell proliferation in a dose-dependent manner (Figure 3A). Studies have reported that IL-35 is primarily produced by Treg cells and that IL-35 executes the functions of these cells. Blocking IL-35 using neutralizing antibodies partially eliminated the immunosuppression of Tregs and restored CD4^+^CD25^−^ T cell proliferation (Figure 3B). These data indicated the IL-35 involved in tumor immunotolerance.

### IL-35 activated the STAT1/STAT3 signaling pathway in human T cells

**Figure 4:**
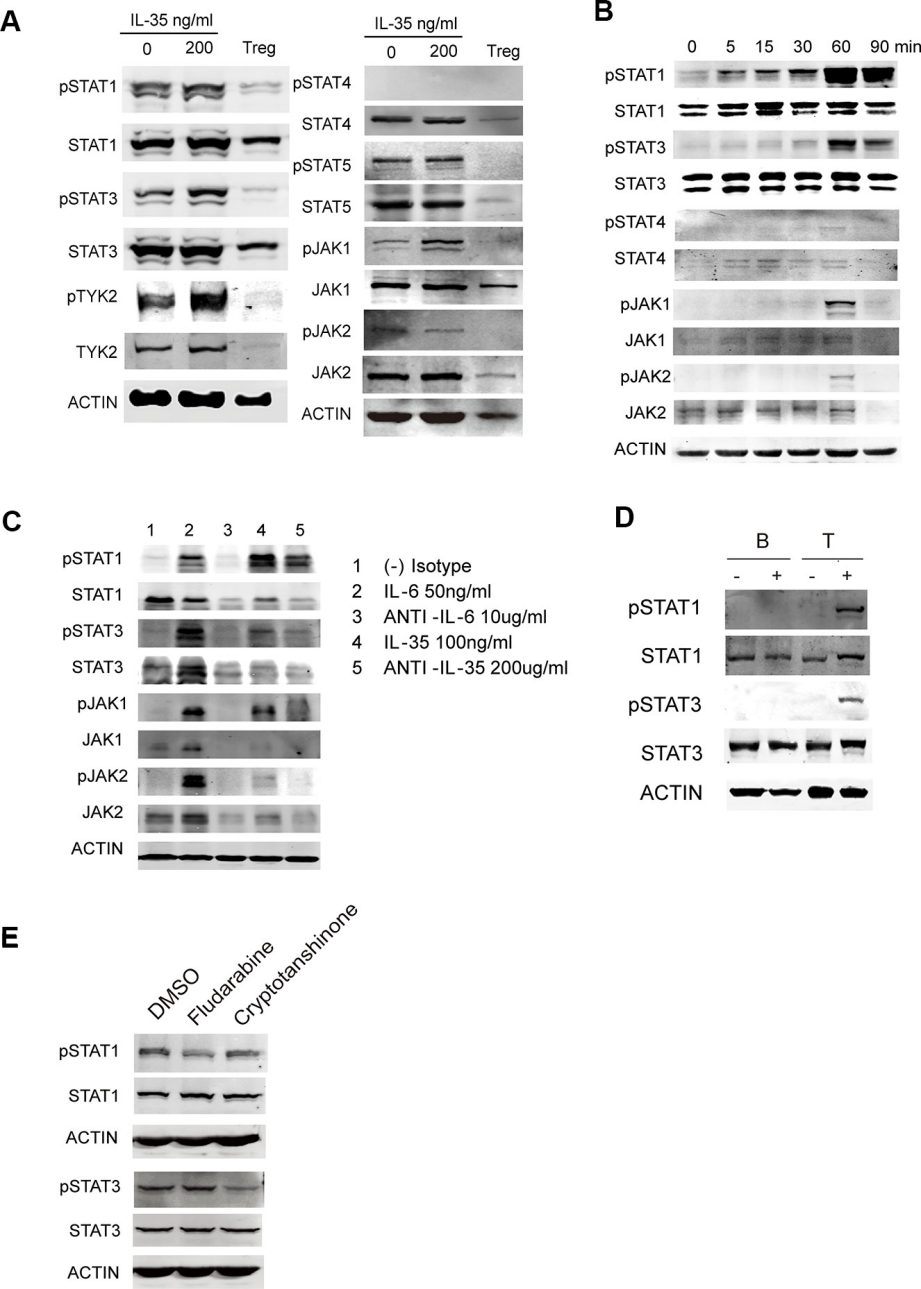
The signaling pathway activated in IL-35-stimulated T cells (**A**) CD4^+^CD25^−^ T and CD4^+^CD25^+^ Treg (1 × 10^5^ per well) cells purified from HDs were activated using αCD3/CD28 with 200 ng/ml rhIL-35 or PBS. Total protein was extracted from cells collected on day 5 and analyzed to determine the levels of IL-12 family-related transcription factors using western blot analysis. (**B**) The phosphorylated and total levels of STAT1, STAT3, JAK1, and JAK2 were examined using immunoblotting assays immediately in 200 ng/ml rhIL-35-activated freshly isolated Pan T cells. (**C**) An IL-35 blockade was applied while the cells were activated with rhIL-35, as described. The phosphorylated and total expression levels of STAT1, STAT3, JAK1, and JAK2 were examined after 60 min. IL-6 blockade was used as the positive control. (**D**) The phosphorylated and total expression levels of STAT1 and STAT3 were examined in purified B and T cells using immunoblotting assays after they were rhIL-35-activated. (**E**) The phosphorylated and total expression levels of STAT1 and STAT3 were examined in PBMCs treated with fludarabine (STAT1 inhibitor, 10 μmol/ml) and cryptotanshinone (STAT3 inhibitor, 20 μmol/ml) and rhIL-35 together for 1 hr. The data shown are representative of three independent experiments.

Studies in mice have reported that IL-35 activates STAT1 and STAT4 heterodimers, or either STAT1 or STAT4 homodimers in T cells (21). While, IL-35 activates STAT3 in B cells (22). To analyze the IL-35 signaling pathway in human T cells, we analyzed a series of transcription factors known to be related to the IL-12 family in CD4^+^CD25^−^ T cells following stimulation with or without IL-35 compared with Tregs. Immunoblotting results showed that IL-35 promoted the phosphorylation of STAT1 and STAT3 and activated upstream phospho JAK1 and TYK2. But STAT4 activation was not detected for both long term and transient IL-35 treatment (Figure 4A). IL-35 significantly increased the phosphorylation of STAT1, STAT3 and JAK1 in a time-dependent manner in freshly isolated pan-T cells (Figure 4B). The increased phosphorylation of STAT1, STAT3 and JAK1 was reduced when IL-35 neutralizing antibodies were added (Figure 4C). Because different STATx were activated in human T cells, we also purified B cells to perform the same experiments. The results showed that IL-35 did not increase the total and phosphorylated levels of STAT1 and STAT3 in human B cells (Figure 4D). To verify the STAT1 and STAT3 activation, PBMCs treated with rhIL-35 together with fludarabine (STAT1 inhibitor, 10 μmol/ml) or cryptotanshinone (STAT3 inhibitor, 20 μmol/ml) were examined STAT1 and STAT3 phosphorylation. The inhibitor treatment could partially downregulated specific phosphorylation (Figure 4E). Collectively, these data suggested that IL-35 activated STAT1 and STAT3 in human T cells.

### IL-35 induced positive feedback promotes increased IL-35 production

**Figure 5:**
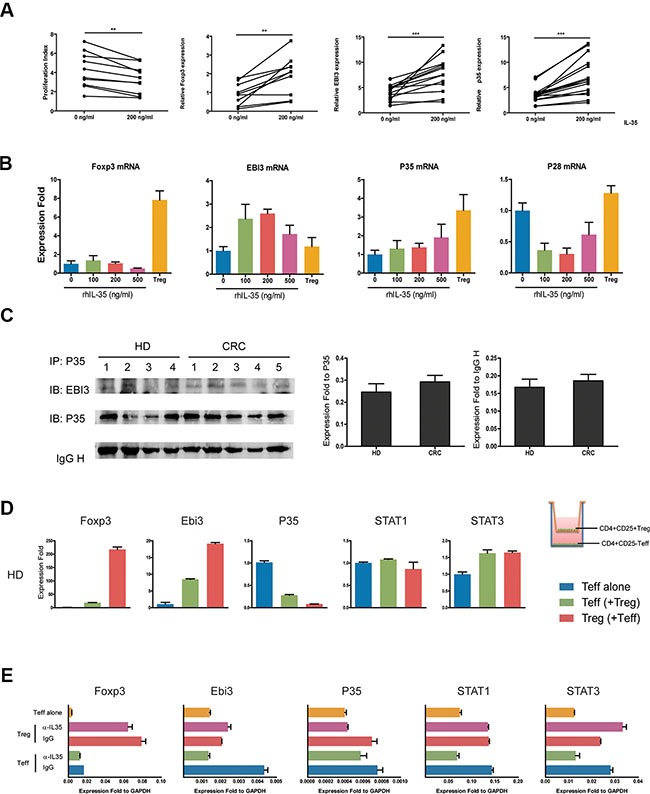
IL-35 production was increased through positive feedback (**A**) CD4^+^CD25^−^ T cells (1 × 10^5^ per well) from HDs (*N* = 10) were incubated with rhIL-35 at a final concentration of 200 ng/ml. Cell proliferation and the expression of Foxp3, EBI3, and P35 mRNAs were observed on day 5. (**B**) CD4^+^CD25^−^ T cells (1 × 10^5^ per well) obtained from HDs were incubated in a series of concentrations of rhIL-35 for 5days, and the expression levels of Foxp3, EBI3, P35 and the IL-12 cytokine family-related subunit P28 mRNAs were analyzed. (**C**) IL-35 expression was analyzed in the culture supernatants of CD4^+^CD25^−^ T cells stimulated with rhIL-35, confirmed following immunoprecipitation with anti-human IL-12A (P35) antibodies, and resolved using SDS-PAGE. The blots were then probed with monoclonal anti-human EBI3 and P35 antibodies. Scan image software detected the gray values of each EBI3, P35 and IgH protein bands. The values of EBI3 were normalized to P35 and IgH, respectively. (**D**) CD4^+^CD25- T cells (1 × 10^5^ per well) obtained from HDs were co-cultured with autologous CD4^+^CD25^+^ T cells (5 × 10^4^ per well) in transwell culture system (0.4 μm) for 5 days. The mRNA expression levels of Foxp3, EBI3 and P35 were observed. Blue bar, HD T_eff_ control which co-cultured with autologous T_eff_; green bar, HD T_eff_ which co-cultured with Treg; red bar, HD Treg which co-cultured with T_eff_. (**E**) IL-35-neutralizing antibodies were added to the coculture systems of CD4^+^CD25^−^ T (1 × 10^5^ per well) cells and autologous CD4^+^CD25^+^ T cells (5 × 10^4^ per well) that were obtained from HDs. After 5 days, the mRNA expression levels of Foxp3, EBI3, P35, STAT1 and STAT3 were observed using Q-PCR. The data shown are representative of three independent experiments. The results are shown as the mean ± SEM. *p < 0.05.

**Figure 6:**
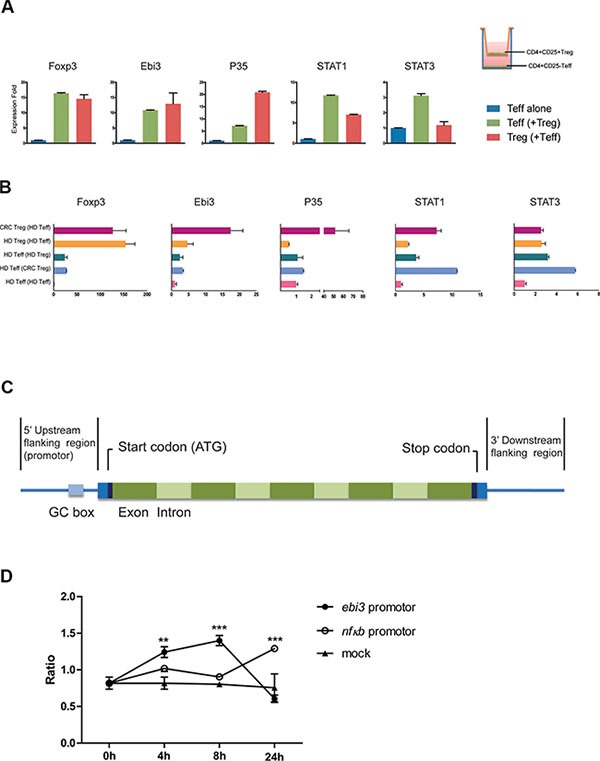
The transcriptional activity of the EBI3 promoter was induced by stimulation with IL-35 (**A**) CD4+CD25^−^ T_eff_ cells (1 × 10^5^ per well) obtained from CRC patients were co-cultured with autologous CD4^+^CD25^+^ Treg cells (5 × 10^4^ per well) in transwell culture system (0.4 μm) for 5 days. The mRNA expression levels of Foxp3, EBI3, and P35 were observed. Blue bar, HD T_eff_ control which co-cultured with autologous T_eff_; green bar, HD Teff which co-cultured with Treg; red bar, HD Treg which co-cultured with Teff. (**B**) CD4^+^CD25^−^ Teff cells (1 × 10^5^ per well) obtained from HD were co-cultured with autologous CD4^+^CD25^+^ Treg cells or allogeneic Treg cells from CRC (5 × 10^4^ per well) in transwell culture system (0.4 μm) for 5 days. Series mRNA expression levels were observed. Violet, CRC Treg which co-cultured with allogeneic T_eff_; orange, HD Treg which co-cultured with autologous T_eff;_ dark green, HD T_eff_ which co-cultured with autologous Treg; lavender, HD T_eff_ which co-cultured with allogeneic T_eff_; pink, HD T_eff_ control. (**C**) Schematic diagram showing the structure of the EBI3 gene. (**D**) An EBI3 promoter-containing fragment (bp −450 to +8 UTR) was ligated into the promoterless luciferase (*luc+*) vector pGL3 basic (Promega). The vectors containing the EBI3 promoter and the NF-κB promoter and the mock plasmids were transferred into human pan T cells using electroporation. After 4, 8 and 24 hours of incubation with 200 ng/ml IL-35, the transcriptional activity of each gene promoter was determined. The data shown are representative of the mean ± SEM. **p < 0.01, ***p < 0.001.

IL-35 has been shown to induce the conversion conventional T cells (Tconv) into IL-35-producing Treg (iTr35) cells, especially in mice. We first purified CD4^+^T cells from 10 healthy individuals, and cells were treated with 200 ng/ml rhIL-35 which promoted the mRNA expression of Foxp3 and IL-35 (EBI3 and P35) (Figure 5A). We used purified CD4^+^CD25^−^ T effectors (T_eff_), CD4^+^CD25^+^ Treg cells as the positive control, and observed that the expression of the EBI3 and P35 mRNAs was increased more than the expression of Foxp3 in CD4^+^CD25^−^ T cells (Figure 5B). Also, IL-10 and TGF-β mRNA was less expressed in CD4^+^CD25^−^ T cells than in Tregs (Supplementary Figure S2A). Immunoprecipitation assays were performed to analyze culture supernatants to confirm the production of IL-35 in CD4^+^ T cells from HD and CRC patients. Results of gray value showed that CD4^+^ T cells from CRC patients produced less IL-35 than HD, but there was no significant difference between two groups (Figure 5C). To determine whether Treg cell were capable of inducing IL-35-producing CD4^+^CD25^−^ T cells, CD4^+^CD25^−^ T_eff_ from HD was co-cultured with autologous Treg for 5 days. After co-cultured with Tregs, T_eff_ markedly increased the expression of Foxp3, EBI3 and P35 mRNAs (Figure 5D and Supplementary Figure S2B). While, IL-35 blockade repressed the expression of EBI3 and STAT1 mRNAs in HD CD4^+^CD25^−^ T cell co-culture systems (Figure 5E). When CD4^+^CD25^−^ T_eff_ from CRC patients were co-cultured with autologous Treg, consistent results could be observed (Figure 6A). To clarify whether Tregs obtained from CRC patients was potential to increase remarkably the expression of Foxp3, EBI3 and P35 mRNAs in T_eff_, HD T_eff_ were incubated with Tregs from HD and CRC using a transwell system. CRC Treg itself expressed higher mRNAs and induced IL-35 related mRNAs expression in HD T_eff_, especially STAT1 and STAT3 (Figure 6B).

To determine whether IL-35 directly participates in the production of IL-35, EBI3 promoter-driven luciferase reporter plasmids and mock plasmids were constructed and transferred into isolated PBMC using electroporation. EBI3 promoter-driven luciferase activity was higher after IL-35 stimulation than the activity induced in cells expressing the mock plasmid (Figure 6C and 6D). Thus, our study indicates that high IL-35 levels in colorectal cancer promotes the increased production of IL-35 via STAT1 and STAT3 and results in the suppression of T cell proliferation, leading to tumor immunotolerance.

## DISCUSSION

Colorectal cancer is a major public health problem in China, where it is currently the second leading cause of cancer death [22, 23]. Tumor immunity and inflammation are regarded as important to carcinogenesis and development in colorectal carcinomas, but the underlying mechanisms of its growth are not yet fully understood. A major question is whether the host's immune system has constitutive house-keeping anti-inflammatory defenses that can counteract the onset of inflammation or whether the host needs to generate and secrete such anti-inflammatory cytokines during inflammation *de novo*.

IL-35 is a newly reported anti-inflammatory cytokine upregulated against the inflammation response, but it is not constitutively expressed as a house-keeping cytokine [24]. IL-35, like IL-12 and IL-27, is a heterodimeric cytokine composed of P35 and EBI3. IL-12 is composed of P35 and p40, and IL-27 is composed of p28 and EBI3 [25, 26]. Although these three cytokines share subunits, their biological functions are very different. IL-12 is a pro-inflammatory regulator of Th1 responses, IL-27 has biphasic functions in inflammation, and IL-35 is inhibitory [27, 28]. EBI3 subunit was considered to be more responsible to its biological functions. Studies in mice have demonstrated that IL-35 plays key roles in autoimmune diseases [11, 18], allergenic diseases [29, 30] and other diseases, such as infection. However, the role of IL-35 in humans requires further study.

Human IL-35 expression has been reported in chronic hepatitis B virus-infected patients [31], normal pregnancies [32], sepsis [33], and rheumatoid arthritis [34]. In support of a mathematical modeling analysis that reported a correlation between IL-35 and tumors [16], we demonstrate that elevated IL-35 level in CRC patients is coincident with an increase in Treg cells and it was mainly expressed in CD4^+^ T cells in human colorectal cancer and adjacent tissues. Its expression in CRC tumor-adjacent tissues is significantly associated with tumor metastasis.

IL-35 as a potent anti-inflammatory cytokine is reported for the suppressive activity of regulatory T cells especially in mice. Our results also shown that reconstituted human IL-35 suppressed proliferation of CD4^+^CD25^−^ T cells, and this suppression could be restored partially using IL-35-neutralizing antibodies. However, we did not observe the considerable rate of inhibition that has been shown *in vitro* in Tregs or in mice studies. It has also been reported that IL-35 specifically attenuates the production of IL-17 in collagen-induced arthritis [11], allergic airways disease in mice [30] or infection [35]. Interestingly, the proportion of Th17 cells in the total population of CD4^+^ T cells in patients with CRC was higher than their proportion in healthy individuals (Supplementary Figure S1A and S1B). Thus, the function of IL-35 in human diseases needs further investigation to determine the mechanisms of its immunoregulation in humans.

Studies have revealed the role of IL-35 in the conversion of murine and human CD4^+^CD25^−^ T cells into IL-35-induced Treg (iTr35) cells [4, 36]. In this work, results showed rhIL-35 could induce the expression of EBI3 and P35 mRNAs, but not p28 or p40 mRNAs, in CD4^+^CD25^−^T cells. It is worth noting that endogenous IL-35 that was derived specifically from CRC patients' Tregs was capable of inducing a further increase in IL-35.

IL-35, by binding to its receptor, recruited and activated of specific members of the STAT family of transcription factors to mediate their biological activities [37, 38]. The IL-35 receptor in mice has been reported to be composed of IL-12Rβ2 subunits (share with IL-12 receptors) and gp130 (share with IL-6 receptors) on T cells [20], or subunit IL-27Rα (share with IL-27 receptors) on B cells [21]. These differences may explain the diversity of downstream STAT-activating activities that have been observed in T and B cells in mice. In this study, we demonstrated that IL-35 treatment activated STAT1 and STAT3 in human CD4^+^ T cells both during incubation and following temporary stimulation. IL-35 did not activate STAT1 and STAT3 in B cells following temporary stimulation. We speculate that IL-35R is not constitutively expressed on human B cells, while is expressed on CD4^+^ T cells in humans. This suggests the possibility that there is diversity in the composition of IL-35 receptors on different cell types or in different species. Meanwhile, the observed differences in the activated signaling pathways indicate that human results may not be reproduced in mice. Thus, when using a genetic deficiency approach to study the role of IL-35 in mice, it might be difficult to obtain consistent data in human diseases. To determine whether IL-35 directly promoted IL-35 production, we constructed an EBI3 promoter-driven luciferase reporter plasmid. After stimulation with IL-35, EBI3 promoter-driven luciferase activity was higher than that of the mock plasmid. EBI3 transcription was reported induced by TLR signaling via NF-κB activation [39]. We used NF-κB promoter-driven luciferase reporter as positive control. Results suggested the indirect links between IL-35 signaling and NF-κB activation.

Taken together, this study demonstrates that elevated IL-35 expression in CRC, especially in tumor-adjacent tissues is significantly associated with tumor metastasis. IL-35 inhibited CD4^+^CD25^−^ T effector cell proliferation *in vitro*, which could be restored partially by applying IL-35-neutralizing antibodies. IL-35 Treatment activated the phosphorylation of STAT1 and STAT3 in human CD4^+^ T cells. IL-35 induced positive feedback to promote IL-35 production, and this process is involved in tumor immunity. Tregs obtained from colorectal cancer patients were more capable of inducing EBI3 and P35 expression than those obtained from healthy individuals. Thus, our study indicates that the high level of IL-35 observed in colorectal cancers promotes its own production via STAT1 and STAT3 to suppress T cell proliferation during tumor immunity. This suggests potential immunoregulatory therapeutic strategies involving IL-35 for the treatment of CRC.

## MATERIALS AND METHODS

### Subjects

Fresh blood and tumor tissues were obtained from 125 sporadic CRC patients who underwent radical resection from March, 2012 to February, 2014 in Xinhua Hospital, Shanghai Jiao Tong University School of Medicine. The patients were histopathologically confirmed to have colorectal cancer. The clinical data and outcomes were obtained from clinical records and pathological reports. All patient characteristics are described in Table 1. We excluded the cases that were treated with radiation, chemotherapy, or immunotherapy before surgery. Patients who had any other history of cancer, autoimmune disease, inflammatory bowel disease, or infectious disease were also excluded. To stage tumors, the sixth edition of the AJCC Cancer Staging Manual was applied (TNM). Patients were divided into two subgroups: TNM I-II and TNM III-VI. The study was approved by the Ethical Committee of Xinhua Hospital affiliated with the Jiao tong University School of medicine. Written informed consent was obtained from all patients, and the study conformed to the principles of the Declaration of Helsinki.

### Blood sampling and flow cytometric analysis

All blood samples used for this study were collected at the time of admission to the department of general surgery. PBMCs were freshly isolated from 4 ml of heparinized blood using Ficoll density gradient centrifugation. Lymphocytes were re-suspended in PBS supplemented with 2% bovine serum albumin at a concentration of 1 × 10^6^ cells/ml. Cell surface marker analysis was performed using four or five color flow cytometric analysis. Fluorochrome-labeled mouse anti-human monoclonal antibodies against CD3-PC7, CD4-PerCP, and CD25-APC were purchased from Beckman Coulter. Foxp3-Ax488 and IgG2a-FITC (eBioscience, CA, USA) antibodies were used in combination with appropriate isotype controls to identify positive and negative cell populations. For double staining of IL-17 and Foxp3, PBMCs were isolated using Ficoll density gradient separation and stimulated with PMA (50 ng/ml) and ionomycin (1 mg/ml) in the presence of Golgi-Stop reagent for 4–6 h. First, extracellular labels were used, including specific Abs against human CD3, CD4, and CD25. The cells were then fixed and permeabilized with Perm/Fix solution and stained using anti-IL-17A (intracellular) and anti-Foxp3 (intranuclear) antibodies or control isotype antibodies for 30 min on ice. Multicolor FACS analysis was performed using a BD FACS Aria flow cytometer (BD).

### Real-time PCR

Total RNA was isolated using Qiagen reagent. Then, first-strand cDNA was synthesized using a Sensiscript RT Kit (Takara) according to the manufacturer's instructions. Real-time RT-PCR was performed as previously described [40]. Data were collected and quantitatively analyzed, and the human GAPDH gene was used as an endogenous control for sample normalization. The following primers were used to assess gene expression: Foxp3, 5′-CTACGCCACGCTCATCCGCTGG-3′ (forward) and 5′-GTAGGGTTGGAACACCTGCTGGG-3′ (reverse); EBI3, 5′-CTTCGTGCCTTTCATAAC-3′ (forward) and 5′-GCTCCCTGACGCTTGT-3′ (reverse); P35, 5′-CTCCTGGACCACCTCAGTTTG-3′ (forward) and 5′-GGTGAAGGCATGGGAACATT-3′ (reverse); P28, 5′-GGAGCTCGTCTTATCTCGGG-3′ (forward) and 5′-TCCAAGGCTGATGATGCGAA-3′ (reverse); P40, 5′-GGCCAGTACACCTGTCACAA-3′ (forward) and 5′-CTGATTGTCGTCAGCCACCA-3′ (reverse); STAT1, 5′-TGTATGCCATCCTCGAGAGC −3′(forward) and 5′-AGACATCCTGCCACCTTGTG-3′ (reverse); STAT3, 5′-ACTTCTTCACTAAGCCGCCA-3′(forward) and 5′-CCATGTGATCTGACACCCTG-3′ (reverse); STAT4, 5′-GAATTGGAGCCCAGTAAGGTC-3′ (forward) and 5′-ATTCCACTGAGACATGCTGGA-3′ (reverse); GAPDH, 5′-ATTCCACCCATGGCAAATTC-3′ (forward) and 5′-GCATCGCCCCACTTGATT-3′ (reverse).

### ELISA, immunoprecipitation, and immunohistochemical and immunofluorescence staining to detect human IL-35

Colorectal cancer, tumor-adjacent tissues and mucosal tissues (200 mg) were lysed in lysis buffer (Beyotime) gently using a MACS Dissociator (Miltenyi Biotec), and protein lysis supernatants were collected for analysis. Serum and tissue lysis supernatants were immunoprecipitated with anti-human IL-12A antibodies (P35 H-197:sc-7925; Santa Cruz) overnight and then coupled to protein A/G-Agarose beads (sc-2003 Santa Cruz) for 4 hours. Immunoprecipitates were resolved using SDS-PAGE, and blots were probed using monoclonal anti-human EBI3 antibodies (EBI3 H-6:sc-365342; Santa Cruz). A human IL-35 ELISA kit (Biolegend) was used to determine the concentration of IL-35 in serum and protein lysates derived from tissues. For immunohistochemical analysis, formalin-fixed, paraffin-embedded clinical tissue sections were prepared and stained using human IL-35 antibodies and a VECTASTAIN Elite ABC kit (Vector Laboratories) according to the manufacturer's protocol. Mouse anti-human IL-35 monoclonal antibodies (1:300, 15K8D10; IMGENEX) was used as the primary antibody. Cells expressing cytoplasmic IL-35 were counted for scoring in lymphocytes. The intensity of immunoreactivity was graded on a scale from 0 (no staining) to 3 (strong immunoreactivity), and the proportion of stained cells (0, no staining; 1, 1–25% staining; 2, 26–50% staining; 3, 51–75% staining; and 4, 76–100% staining) was determined. The total score was determined using the staining intensity plus the percentage of positive cells as follows: low expression group (total score < 1) and high expression group (total score ≥ 1) to better analyze prognoses between groups. All slides were evaluated by two pathologists. For immunofluorescence staining, colorectal tissue frozen sections were stained with anti-IL-35 (1:100) and anti-CD4 (1:100, Santa Cruz, CA, USA) primary antibodies. They were then exposed to Alexa Fluor 568/488-conjugated secondary antibodies and counterstained using DAPI. Slices were visualized using a fluorescence microscope (Olympus Corp, Tokyo, Japan).

### *In vitro* cell culture

For cell sorting, 10 ml of peripheral blood was collected from either HDs or patients, and CD4^+^ T cells and CD4^+^CD25^−^ T cells were obtained from PBLs using a human CD4^+^ T Cell Isolation Kit II and a CD4^+^CD25^+^ Regulatory T Cell Isolation Kit (Miltenyi Biotec), respectively. The purity of the cells was 90% or greater, as determined by repeated analysis. The cells were incubated with rhIL-35 (Sino Biological Inc., Beijing, China) at a final concentration 100 nM or in PBS, which was used as the control, and stimulated with pre-coated 5 μg/ml αCD3, soluble 5 μg/ml αCD28 and 200 U/ml rhIL-2 at 1 × 10^5^ per well in 96-well U-bottom plates. Three replicates were performed for each condition. The cells were cultured in a humidified CO_2_-containing atmosphere at 37°C for 5–7 days in complete RPMI-10 medium supplemented with 100 U/ml penicillin, 100 μg/ml streptomycin, 0.5 mM sodium pyruvate, 0.05 mM nonessential amino acids, 2 mM L-glutamine, and 10 mM HEPES (all from GIBCO). Inducible IL-35-secreting regulatory T cell culture experiments were performed using a transwell co-culture system (0.4 μm). A mouse monoclonal antibody for IL-35 (V1.4F5.25, Shenandoah) was used to functionally block rhIL-35. For cell proliferation experiments, purified T cells were stained with CFSE, and cell division was detected using flow cytometry after 5 days of incubation.

### Western blot analysis

Total protein was extracted from PMBCs or CD4^+^ T cells that were cultured with rhIL-35 or anti-IL-35-neutralizaing monoclonal antibodies. Cell homogenates were boiled, and the proteins were separated using SDS-PAGE. After overnight incubation at 4°C with anti-phospho-STAT1 (Tyr701, 58D6), anti-STAT1 (9H2), anti-phospho-STAT3 (Tyr705, D3A7), anti-STAT3 (124H6), anti-phospho-STAT4 (Tyr693, D2E4), anti-STAT4 (2A2), anti-phospho-STAT5 (Tyr694, D47E7), anti-STAT5 (3H7), anti-phospho-JAK1 (Tyr1022/1023), anti-JAK1 (6G4), anti-phospho-JAK2 (Tyr1007/1008, C80C3), or anti-JAK2 (D2E12) antibodies (all Cell Signaling Technology, Beverly, MA), the membranes were incubated with IRDye 800 goat anti-rabbit or IRDye 680 goat anti-mouse secondary antibodies (both LI-COR Biosciences, Lincoln, NE, USA). The targeted proteins were detected and quantified using a Li-COR Odyssey infrared imaging system (LI-COR Biosciences).

### EBI3 promoter activity detected using luciferase assays

A fragment containing the EBI3 promoter (bp −450 to +8 UTR with respect to the transcription initiation site) was ligated into the promoterless luciferase (*luc*+) vector pGL3 basic (Promega) (Supplementary Table S1 and Supplementary Figure S3A and S3B). The NF-κB luciferase reporter plasmid was a gift from Dr. Guohua Xie. All constructs used in this study were verified using restriction map and sequencing analyses. A total of 2 mg of the EBI3 promoter-containing, NF-κB promoter-containing and mock plasmids were transferred into human pan T cells using electroporation. The efficiency of transfection into primary cells was shown by GFP expression in fluorescency microscopy (Supplementary Figure 3C). After 4 h, 8 h and 24 h hours of incubation with 200 ng/ml IL-35, the transcriptional activity induced by each gene promoter was determined using a Dual-Luciferase Reporter Assay System (Promega) according to the manufacturer's instructions.

### Statistics

Statistical evaluations were performed using GraphPad Prism (version 5.0). Values are shown throughout the manuscript as the mean ± SEM except for the patients and the HD ages, which are shown as the mean ± SD. Student's *t*-tests were used to analyze the differences between the groups, and one-way ANOVA was initially performed to determine whether an overall statistically significant difference was present before two-tailed paired or unpaired Student's *t*-tests were used for normally distributed data. In cases where there was a significant difference between subgroups, post hoc analyses were performed based on the Tukey test (for normally distributed data) or the Mann-Whitney *U-test*. Kaplan-Meier survival curves were plotted, and log rank tests were performed. The significance of various variables related to survival was analyzed using the Cox proportional hazards model in a multivariate analysis. A *p value* of < 0.05 was considered to be statistically significant.

## SUPPLEMENTARY MATERIALS TABLE FIGURES


