# Supplementary material for: Elevated level of Interleukin-35 in colorectal cancer induces conversion of T cells into iTr35 by activating STAT1/STAT3
Source: Oncotarget. 2016 Sep 22;7(45):73003–15. doi: 10.18632/oncotarget.12193 (PMC5341959; doi:10.18632/oncotarget.12193)
Supplement: Supplementary file 1 [file oncotarget-07-73003-s001.pdf]

Elevated level of Interleukin-35 in colorectal cancer induces conversion of T cells into iTr35 by activating STAT1/STAT3

Supplementary Materials

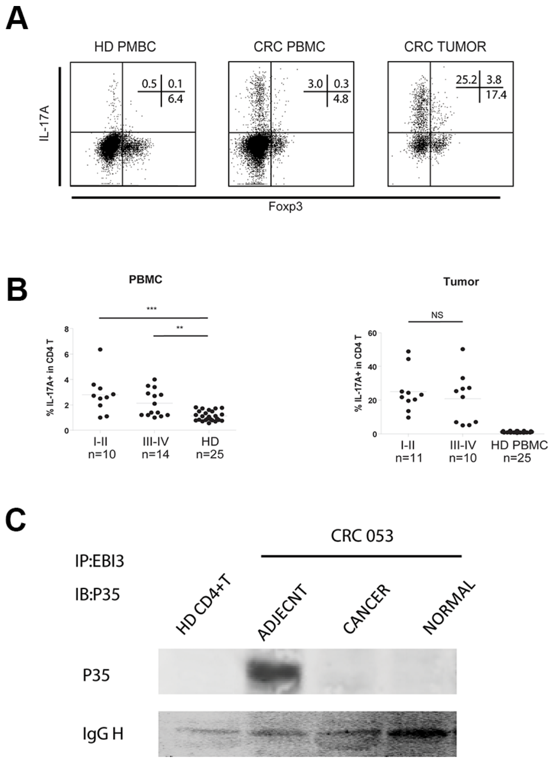

Supplementary Figure S1: IL-17 and P35 expression in CRC. (**A**) Representative flow cytometric (FCM) dot plots showing IL-17 and Foxp3 double-labeled CD4 T cells both in healthy donors (HD) and CRC patients. (**B**) Proportions of Treg cells in CRC patients in early and advanced clinical stages compared to the proportions in healthy donors. (**C**) (200 mg) lysis supernatants were immunoprecipitated using anti-human EBI3 antibodies and then coupled to protein A/G-agarose beads. Proteins were resolved using SDS-PAGE, and the blots were then probed using monoclonal anti-human P35 (IL-12A) antibodies.

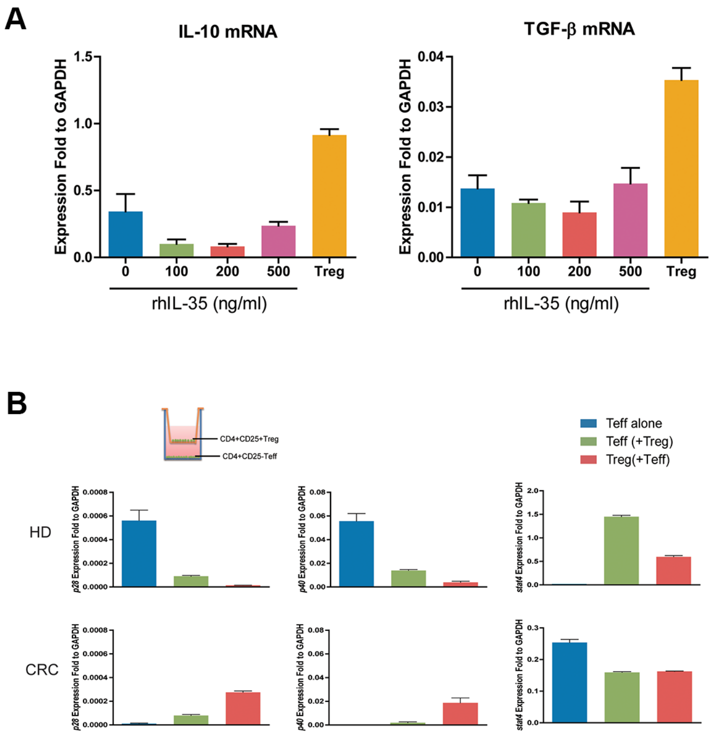

Supplementary Figure S2: The mRNA expression of related cytokines and transcriptor. (**A**) CD4+CD25− T cells (1×10^5 per well) obtained from HDs were incubated in a series of concentrations of rhIL-35 for 5 days, and the expression levels of IL-10 and theTGF-β mRNAs were analyzed. (**B**) $CD4^+CD25^-$ T cells (1×10^5 per well) obtained from HD and CRC were co-cultured with autologous $CD4^+CD25^+$ T cells (5×10^4 per well) in transwell culture system (0.4 µm) for 5 days. The mRNA expression levels of P28, P40 and STAT4 were observed. Blue bar, HD T_{eff} control which co-cultured with autologous $T_{eff;}$ green bar, HD T_{eff} which co-cultured with Treg; red bar, HD Treg which co-cultured with T_{eff}.

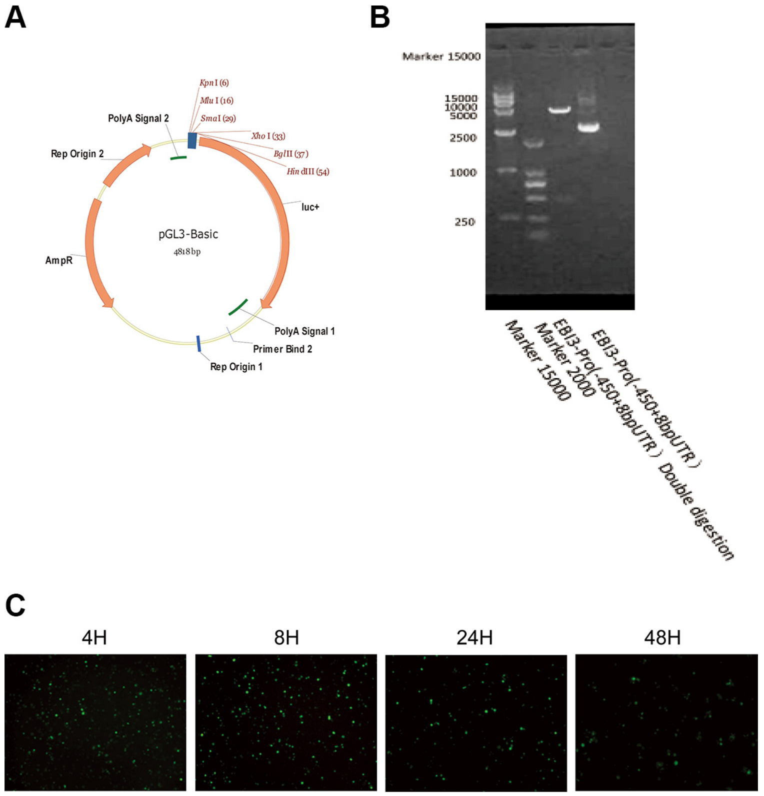

Supplementary Figure S3: Construction of luciferase reporter gene vector for human EBI3 gene promoter. (**A**) The information of pGL3-basic. (**B**) Verification of EBI3 promoter by Double digestion and sequencing. (**C**) Transfection efficiency of GFP plasmid observed under transfection efficiency of plasmid.

Supplementary Table S1: Primers of luciferase reporter gene vector for human EBI3 gene promoter

	**Primer 5′to 3′**
EBI3-Pro(−450+8bpUTR -F(MluI)	CCGG ACGCGT TGTCTTCCTTCTGTCTTCTCTGTCTCTCTGC
EBI3-Pro(−450+8bpUTR -R(HindIII)	GCCC AAGCTTGGCTGCGGCCAGCTCTGC
